# Data sources for precision public health of obesity: a scoping review, evidence map and use case in Queensland, Australia

**DOI:** 10.1186/s12889-022-12939-x

**Published:** 2022-03-24

**Authors:** Oliver J. Canfell, Kamila Davidson, Clair Sullivan, Elizabeth Eakin, Andrew Burton-Jones

**Affiliations:** 1 UQ Business School, Faculty of Business, Economics and Law, The University of Queensland, St Lucia, QLD Australia; 2 Centre for Health Services Research, Faculty of Medicine, The University of Queensland, Herston, QLD Australia; 3 Digital Health Cooperative Research Centre, Australian Government, Sydney, NSW Australia; 4 Health and Wellbeing Queensland, Queensland Government, The State of Queensland, Milton, QLD Australia; 5 Department of Health, Metro North Hospital and Health Service, Queensland Government, Herston, QLD Australia; 6 School of Public Health, Faculty of Medicine, The University of Queensland, Herston, QLD Australia

**Keywords:** Public health, Public health informatics, Medical informatics, Obesity, Noncommunicable diseases, Decision making

## Abstract

**Background:**

Global action to reduce obesity prevalence requires digital transformation of the public health sector to enable precision public health (PPH). Useable data for PPH of obesity is yet to be identified, collated and appraised and there is currently no accepted approach to creating this single source of truth. This scoping review aims to address this globally generic problem by using the State of Queensland (Australia) (population > 5 million) as a use case to determine (1) availability of primary data sources usable for PPH for obesity (2) quality of identified sources (3) general implications for public health policymakers.

**Methods:**

The Preferred Reporting Items for Systematic Review and Meta-Analyses extension for scoping reviews (PRISMA-ScR) was followed. Unique search strategies were implemented for ‘designed’ (e.g. surveys) and ‘organic’ (e.g. electronic health records) data sources. Only primary sources of data (with stratification to Queensland) with evidence-based determinants of obesity were included. Primary data source type, availability, sample size, frequency of collection and coverage of determinants of obesity were extracted and curated into an evidence map. Data source quality was qualitatively assessed.

**Results:**

We identified 38 primary sources of preventive data for obesity: 33 designed and 5 organic. Most designed sources were survey (*n* 20) or administrative (*n* 10) sources and publicly available but generally were not contemporaneous (> 2 years old) and had small sample sizes (10-100 k) relative to organic sources (> 1 M). Organic sources were identified as the electronic medical record (ieMR), wearables, environmental (Google Maps, Crime Map) and billing/claims. Data on social, biomedical and behavioural determinants of obesity typically co-occurred across sources. Environmental and commercial data was sparse and interpreted as low quality. One organic source (ieMR) was highly contemporaneous (routinely updated), had a large sample size (5 M) and represented all determinants of obesity but is not currently used for public health decision-making in Queensland.

**Conclusions:**

This review provides a (1) comprehensive data map for PPH for obesity in Queensland and (2) globally translatable framework to identify, collate and appraise primary data sources to advance PPH for obesity and other noncommunicable diseases. Significant challenges must be addressed to achieve PPH, including: using designed and organic data harmoniously, digital infrastructure for high-quality organic data, and the ethical and social implications of using consumer-centred health data to improve public health.

**Supplementary Information:**

The online version contains supplementary material available at 10.1186/s12889-022-12939-x.

## Background

Global public health attempts to address obesity are failing [1]. Obesity is entirely preventable; yet to date, no country has been successful in reversing its growing prevalence, which has nearly tripled since 1975 [2]. Obesity is a chronic, relapsing, progressive disease process that is caused by complex, interwoven and systemic interactions between social, biomedical, behavioural, environmental and commercial determinants of health [3–5]. We need new ways of working that challenge and transform traditional models of public health into a predictive, preventive and responsive ‘learning’ system.

Decision-making by public health actors has always relied upon population data. The problem with this decision model is that data is captured retrospectively and is thus outdated before it is available to decision-makers, and disease prevalence, incidence and risk factors are typically captured as cross-sectional snapshots. This risks ineffective public health decision-making as decisions are made upon old information. Rapid technological advancement – particularly the rise in the Internet of Things (IoT) in a health and medical context – has generated an explosion of data*,* an asset that has been coined the new oil of the twenty-first century [6]. This organic, routinely collected data is beginning to transform the public health sector and stakeholder decision-making by providing the foundations for precision public health (PPH). PPH is a modern reconceptualization of traditional public health that uses routinely collected data and digital technology to guide precision decisions, practice and policy to improve population health [7, 8]. Specifically, PPH aims to improve *measuring* and *targeting* of the determinants of health at a systems level with a focus on health equity to deliver the right intervention to the right population every time.

With limited mature and evidence-based examples of PPH for obesity prevention in practice, we offer an exemplar here: community public health units access a digital dashboard containing (1) retail transactional data for fast food that is geotagged with hotspots in real-time (2) weight trajectory risk data in the early years leveraged from a statewide electronic medical records (EMR) (3) census data that augments geotagging with understanding of social position e.g. education, household income and the built environment. Then by harnessing a richly curated digitally connected community, public health professionals can ‘push’ in real-time, at the right time, digital messages and content via social media and mHealth applications to promote healthy, family-friendly alternatives that are low cost and readily available to individuals in high-risk areas and monitor their impact. This example of PPH for obesity prevention contributes to a responsive learning *public* health system, where all data entered is used to improve prevention and policy decisions for future consumers, populations and the public [9]. This is only realised if decision-makers can access and interpret rich, contemporaneous, high-quality data across all determinants of obesity – social, environmental, behavioural, biomedical and commercial – to direct precision interventions, resourcing, policy and investment.

There is significant opportunity to transform the public health model for obesity prevention towards PPH by harnessing rich, aggregated data across all its determinants. As healthcare is shifting towards using more contemporary, routinely collected data to continuously improve decisions, public health must be concurrently agile and action a responsibility to use a deeper array of data for better decisions. The inevitable disruption caused by this digital transformation of the public health sector cannot be underestimated. Traditional public health workflows, decision-making processes, skill capabilities and outcome measures will change. This digital disruption has been heavily researched and quantified in the digital transformation of the acute healthcare sector, primarily with the implementation of EMRs and the ‘digital hospital’ era [10, 11].

As a first step to realising PPH for obesity, useable data must be identified, collated and appraised. There is also currently no universally accepted approach to creating and assessing a single source of truth of data for PPH of obesity. To address these gaps, we will leverage the use case of Queensland, Australia to exemplify a solution to these globally generic problems. Queensland has a population > 5 million and is geographically two times the size of Texas and five times the size of Japan [12]. Queensland is well-positioned to advancing PPH for obesity with recent statewide investment in health promotion and digital health; it is a generalisable case that international jurisdictions can emulate. Therefore, the aims of this scoping review are:

1. To determine the availability of primary sources of data useable for PPH for obesity in Queensland, Australia
2. To assess the quality of identified primary data sources
3. To explore the general implications of (a) our findings and (b) digital transformation of the public health sector for public health agencies and policymakers

We hypothesise that data useful for obesity prevention exists but in siloed, fragmented pockets across sectors and that commonly used data sources are highly accessible but poor quality. In leveraging Queensland as a generalisable population use case, our intent is to propose a globally re-usable framework for identifying, collating, and appraising primary data sources for PPH of obesity and other noncommunicable diseases (NCDs).

## Method

### Definitions

#### Designed and organic data

There are two primary types of data: designed and organic [13]. Designed data is created using specialised methods designed by the user, often as single cross-sectional snapshots, including point-prevalence surveys, administrative databases and disease registries [13]. This data provides the cornerstone of traditional public health decision-making and policy. Global examples of designed data include: Australia (National Health Survey, Census), USA (Behavioural Risk Factor Surveillance System), Japan (National Health and Nutrition Survey).

Organic data is routinely collected without design [13]. Organic data relevant to public health can be sourced from electronic medical/health records (EMRs/EHRs), personal health records (PHRs), insurance claims and billing databases, mobile health (mHealth) applications, wearables and sensors (IoT), social media, telecommunications and commercial transactions, as examples.

Organic data offer numerous advantages to designed data in the context of obesity prevention: it can provide measurement of social, environmental, biomedical, behavioural and commercial determinants of health in a frequency that is near real-time, offer high geospatial reach in underserved and priority areas, target the early and adolescent years (when obesity prevention and intervention is most effective) via standardised entry points to the health system and facilitate social technology integration (e.g. mHealth, wearables, social media).

#### Determinants of obesity

Table 1 outlines determinants of health relevant to obesity prevention [14–18].

**Table 1:** Determinants of health relevant for obesity prevention

Level I Determinant of obesity	Level II Sub-determinant of obesity	
Social	Housing	Employment
	Income	Education
	Marital status	Family size/structure
	Ethnicity	Religion
	Child development	Domestic violence and personal safety
Biomedical	Sex^R^	Chronic diseases
	Weight^R^, height^R^, weight status^C^	Age^R^
	Waist circumference^R^	Body Mass Index (BMI)^C^
	Disability	Blood pressure^R^
	Birth weight^R^	Maternal weight status (BMI)^C^
	Rapid infant weight gain^C^	Mental health
	Family medical history	Medication
Behavioural	Dietary patterns	Physical activity
	Breastfeeding	Smoking
	Sleep	Alcohol consumption
	Parenting practices	Responsive feeding
Environmental	Geographical location (rurality)	Transport system
	Food availability	Time commuting
	Access to technology/Internet access/connection	Access to health services
	Green and open spaces	Density and type of food outlet
	Residential density	Geographical location/crime
Commercial	Marketing	Food advertising
	Food processing	Nutrition labelling
	Affordability	Price Index

*BMI* Body Mass Index

^R^raw elements

^C^computed elements

### Design

We conducted a scoping review due to the exploratory nature of our research questions and aims [19]. A scoping review design is flexible in the type of evidence that can be included and consequently it has been proposed as an ideal evidence assessment approach for digital health [20]. We adhered to the Preferred Reporting Items for Systematic Reviews and Meta-Analyses extension for Scoping Reviews (PRISMA-ScR) checklist (see supplementary file 1) [21]. Our review protocol was not published as PROSPERO does not accept protocols for scoping reviews.

We modified the approach of Peters et al. [22] to prioritise data sources rather than empirical literature in the following steps:

1. Creating research questions using the Population, Concept, Context (PCC) criteria (Table 2)
2. Determining the selection criteria and search strategy
3. Designed and organic data source searching and information extraction
4. Mapping designed and organic data sources against the determinants of obesity
5. Conduct a gap analysis in data sources for specific determinants of obesity
  a Approach subject matter experts (SMEs) to query gaps in data sources
  b Targeted searching for identified gaps
6. Critical appraisal of designed and organic data sources quality
7. Reporting results

**Table 2:** Population, Concept and Context (PCC) criteria for the current scoping review

Domain	Criteria
Population	All ages
Concept	Primary sources that contain designed and organic data on the determinants of obesity
Context	Australia (data stratified to Queensland, Australia) The State of Queensland, Australia (data specific to Queensland, Australia)

### Search strategy

A unique search strategy was developed for (a) designed and (b) organic data sources. A framework for the determinants and sub-determinants of obesity was used as the evidence-based pillar to guide search strategy development, data extracting and data mapping (Table 1). Searches were performed by one researcher (KD) and iteratively discussed with another researcher (OJC).

#### Designed data sources

1. Collated key published data reports
  Data reports on the health of the Queensland population were identified from expertise of the research team. These reports presented aggregate data from national health surveys stratified to Queensland [23, 24] and health needs assessment data from Primary Health Networks [25–30].
2. Conducted literature search
  The literature searches were co-designed with a university research librarian.
  (a) Grey literature search
  Keywords ‘obesity’ and ‘Queensland’ were used to search the platforms Google and Google Scholar for data sources for each Level I determinant of obesity (see supplementary file 2 for one sample search strategy). We searched the first 10 pages of Google and Google Scholar that displayed 10 results per page.
  (b) Scholarly literature search
  The databases Embase, Scopus and Web of Science were searched in July 2021. A range of keyword combinations were used including ‘obesity’, ‘Queensland’ and/or each separate domain of obesity determinants (e.g. ‘physical activity’).
3. Identified primary data sources
  Primary data sources were identified by one author (KD) searching the reference lists of key data reports and snowballing when necessary until the primary data source was identified. Relevant government websites that host statistical population health data (e.g. Australian Bureau of Statistics, Queensland Open Data Portal) were interrogated for primary data sources.
4. Identified gaps in designed data sources for determinants of obesity
  Data from primary designed data sources were mapped to the Level I and Level II determinants of obesity. One author (KD) conducted a data gap analysis for each Level II sub-determinant of obesity.
5. Engaged external stakeholders and SMEs
  External organisational stakeholders in public health, academia and healthcare, academic and health system SMEs and local council were contacted to ask for any known designed data source that could fill identified data gaps, particularly at a community and population group level (e.g. via a preventive health program, clinical practice or council dataset). All recommended reports and primary data sources arising from engagement were interrogated.

#### Organic data sources

##### Clinical

Two organic data sources were identified as health records: the statewide Queensland integrated-electronic medical record (ieMR) and national My Health Record (MHR) (electronic/personal health record). The ieMR services ~ 70% of acute public hospital services in Queensland and collects data on every patient who interacts with a digital hospital. MHR is an opt-out national (Australian Government) electronic (personal) health record which operates within tight national legislative and governance controls [31]. MHR users control their health information and can delegate permissions to their health professionals as required, and can choose to share their MHR data for public health and research purposes [32]. We decided to primarily interrogate the ieMR as most relevant to this review.

Two strategies were implemented to gather necessary information about ieMR. Firstly, government websites were searched for information regarding ieMR, specifically details about the collected data that is relevant to the determinants of obesity. Secondly, we investigated the data elements routinely collected via publicly available information.. Data were then mapped against the determinants of obesity.

##### mHealth

Three private/industry websites, Garmin, Fitbit and Apple, were searched for relevant digital health wearables after consultation with mHealth SMEs. These websites were searched for information on data collected from consumers via digital health wearables. The most popular digital health wearable was selected from each company as an exemplar [33] and data on determinants of obesity was extracted and mapped.

##### Billing and claims

Data on billing and claims was accessed via Medicare Statistics (Medicare Australia), Australia’s universal health scheme [34, 35]. Medicare Item Reports allows a user to produce reports on listed Medicare services and pharmaceuticals subsidised by the Australian Government [35]. Medical Benefits Schedule (MBS) and Pharmaceutical Benefits Scheme (PBS) items and their detailed description can be found via MBS website [36] and PBS website [37], respectively. Once specific item numbers are known these can be entered into the Medicare Statistics website to run statistics and reports on their use within Queensland.

## Eligibility criteria

We included primary data sources that host designed and organic data on the determinants of obesity according to the eligibility criteria described in Table 3. We did not define data reports as primary data sources as they present secondary aggregation and reporting of data. We considered raw (e.g. weight, height), computed (e.g. BMI), temporal (e.g. infant weight gain) and non-temporal (e.g. sex) data.

**Table 3:** Eligibility criteria for designed and organic data sources in the present scoping review

	**Inclusion criteria**	**Exclusion criteria**
Population	All ages	Nil
Concept	Data sources: • Contain data on the determinants of obesity • Report on their availability • Curated between years 2000–2021 • Data can be stratified by State • Data describe any single or collective determinant/s of obesity	• Data from traditional clinical research settings (i.e. RCTs, interventional trials) • Data from States or Territories in Australia other than Queensland • Data from an international source
Context	State of Queensland, Australia • Data reported at a state and/or regional, community and individual level	• Data from States or Territories in Australia other than Queensland • Data from an international source

*RCT* Randomised controlled trial

## Data extraction, mapping and synthesis

Data associated with Level I and Level II determinants of obesity (Table 1) were extracted and mapped to their specific determinant. The following characteristics of data sources were extracted: data owner/organisation, name of data source, design type (survey, administrative data, registry, index, EMR, mHealth and geographical), availability, sample size, latest collection and frequency of collection (supplementary file 3). Data availability was reported as public (freely available or by request) or governed, and noted if there is a cost associated with access. We compiled an evidence map for designed and organic data sources as a single source of data truth for prevention and public health decision-making for obesity.

## Quality assessment

Quality of data sources was appraised according to six domains aggregated from two evidence-based tools for assessing data quality in administrative [38] and big data [39]: accessibility (cost and ease to access), usability (data file formats), age (latest collection and frequency of repeat) and timeliness of availability (delay from data generation to utilisation), granularity (richness and details reported) and data coverage of determinants of obesity.

## Results

We identified 38 primary data sources; 33 sources of designed data [40–73] and five sources of organic data: ieMR [74], digital health wearables [75–77], Google Maps [78], state crime map [79] and billing/claims [34, 35]. Supplementary file 3 includes all extracted information on each data source, including their coverage of each Level I and II determinant of obesity.

**Fig. 1:**
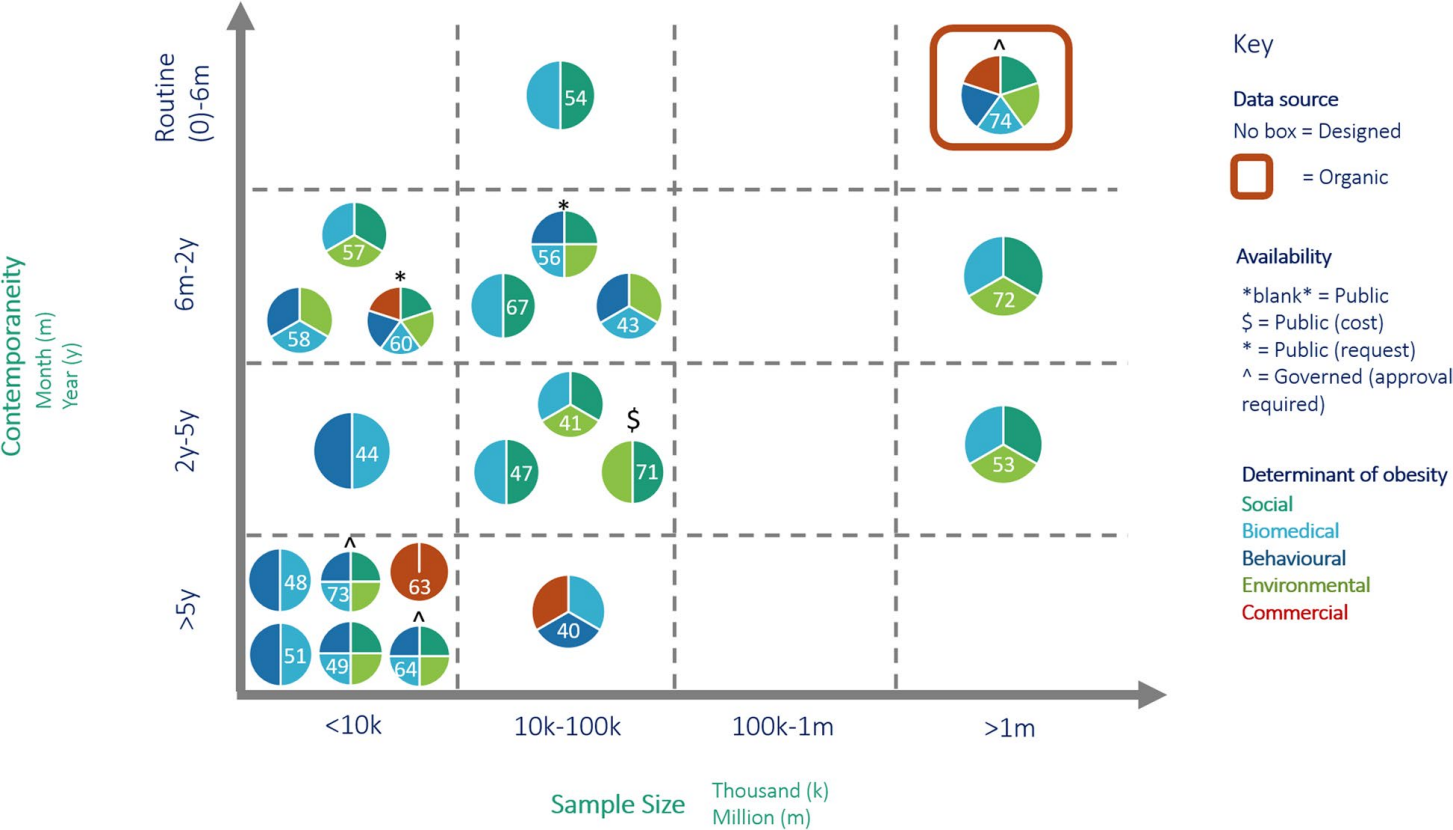
Key characteristics of primary sources of designed (*n* = 20) and organic (*n* = 1) data with complete information. Each primary source is referenced inside each pie chart

Figure 1 presents a visual representation of data contemporaneity, data source sample size and coverage of Level I determinants of obesity for each designed and organic data source with complete information available (*n* 21) – sources closer to the upper right quadrant indicate higher usability for PPH for obesity. The remaining data sources (*n* 17) had missing sample size data – source details are provided in supplementary file 3 and are evaluated qualitatively in the below sections.

Overall, most primary sources of designed data are concentrated in the lower left quadrants of Fig. 1, indicating reduced contemporaneity and sample size. Most designed data are publicly available. Two groups (1) social, biomedical and environmental and (2) biomedical and behavioural determinants of obesity typically co-occur in designed data sources. Commercial determinants of obesity are the least represented. One primary source of organic data (ieMR [74, 80]) is presented in the upper right quadrant and demonstrates complete coverage of all determinants of obesity with governed access.

### Availability of data sources

#### Designed data

The final list comprised 33 primary sources containing data on determinants of obesity (supplementary file 3). Twenty-three sources are owned by the Australian Government (Bureau of Statistics, Institute of Health and Wellbeing, National Disability Insurance Scheme), eight by Queensland (state) Government, and two by independent organisations (Melbourne Institute, National Centre for Vocational Education Research Data). We identified 20 surveys, ten administrative data sets and two indexes and one registry. Twenty-eight data sources were publicly available (two required a request submission and one involved a cost for some datasets) and five were governed.

Figure 1 shows eight data sources hosted sample sizes of < 10 000, eleven sources of 10 000–100 000, nil sources of 100 000-1 M, two sources of > 1 M and 13 sources with no sample size data available. Six data sources collected data at a frequency of 0–6 months, 14 at 6 months-2 years, five at 2–5 years and eight at > 5 years.

#### Literature search results

The results were overwhelming and not specific to our research question. Upon discussions with the research team, it was decided that data derived from controlled research studies was outside the scope of this review, and we were to instead focus on in-depth search of primary data sources using methods outside of bibliographic databases. The main reason for this was that scholarly articles often publish research data from targeted cohorts and seek to answer specific research questions. This is less relevant to PPH than designed or organic data sources as data collected has greater diversity and is often collected in large population-representative samples.

#### Organic data

We identified five organic primary data sources (Table 4, supplementary file 3). One organic primary data source (ieMR [74, 80]) is represented in Fig. 1 in the upper right quadrant as highly contemporaneous (routinely collected) with a large sample size (~ 5 m) and coverage of all Level I determinants of obesity.

**Table 4:** Primary sources of organic data relevant to the determinants of obesity

#: Data owner/ organisation: Data source name ^(design type & availability)^	Sample size	Frequency of collection
Queensland Health: ieMR ^EMR, Gov^	~ 5 M	Routine
Garmin, Fitbit, Apple: Digital health wearables ^mH, Gov^	n/a	Routine
Google: Google Maps ^G, Pub^	n/a	Daily-3yrs
Queensland Police: Crime Map ^A, Pub^	n/a	Daily
Australian Government. Department of Health: Billing/claims (MBS and PBS) ^A, Pub^	n/a	Routine

*EMR* Electronic medical records, *mH* mHealth, *G* Geographical, *A* Administrative data, *Gov* Governed, *Pub* Public, *n/a* Not available

Three sources are Government-owned (ieMR [74, 80], Crime Map [79] and billing/claims [36, 37]) and two are privately owned: digital health wearables (Garmin, Fitbit, Apple) and geographical (Google). There are two administrative data types (Crime Map and billing/claims), one EMR (ieMR), one mHealth (digital health wearables) and one geographical data (Google Maps). Data is routinely collected and available instantly with the exception of billing/claims, which is currently available for July 2021.

### Quality of data sources

#### Accessibility

Twenty-eight designed data sources are freely available to the public (e.g. online download), two of which were available upon request and one stipulated that fees may apply for some data sets. Depending on the type of data request, ethical approval may be required to access data for research purposes.

Data extracts and views are freely available at no cost from three organic data platforms (Google Maps, Crime Map and billing/claims). Google Maps offers purchasable products (e.g. Javascript and API solutions) to help users visualise static or dynamic geospatial aggregate data for their projects.. Two (ieMR and digital health wearables) require data request approval from relevant governance structures, which may present lengthy (up to 12 months) wait times. Fitbit requires its devices to be purchased from their website and a research application to be approved by its governance structure [81]. Once approved, access to data is available via a public web API at no charge or via third party platform, ‘Fitabase’ which attracts a fee (dependent on circumstances). Garmin asks for an application to be completed when seeking access to its data which is offered through Garmin Health API or Software Developer’s Kits (SDKs). No details regarding access cost are provided despite specifying that commercial use of data via SDK is subjected to a license fee. Garmin data can also be accessed via ‘Fitabase’. Apple offers a free research app to allow consumers to participate in research and a free ‘ResearchKit’ (open-source software framework) to assist researchers in designing, conducting and collecting data on specific research studies using Apple devices. Only Apple users can access the Research app and volunteer to participate in Apple-supported study.

#### Usability

Twenty-five primary sources of designed data offer data in downloadable Microsoft Excel tables (i.e. government sources), two in zip files which include data files in SAP, SPAA or STATA formats [56, 60] and one in Access file format [57]. State (Queensland) government health data [58] is also available to download as image, PDF file or PowerPoint from the Queensland Survey Analytic System (QSAS) visualization tool.

ieMR data extraction must be performed by specialized and approved government personnel hence the format in which data can be presented could be negotiable. Data from private businesses and organic data sources can be accessed in Excel (Fitbit, and Garmin if accessed via ‘Fitabase’) or JSON format (Garmin) [82]. Apple did not publish details about its data files. Aggregete data from the Queensland Police Crime map can be exported as pdf or Excel files. Google Maps allows users to download personal data once a Google account is created. The user can save and export selected data from Google Maps in the Keyhole Markup Language (KML) format (a Google-owned type of Geographic Information System (GIS) format). Aggregate data for billing/claims is available in report format which can be downloaded to an Excel spreadsheet.

#### Data age and timeliness of availability

Designed data sources were not routinely collected and typically collected data at a frequency of 1 year or greater (*n* 27 sources). The time from data collection to data availability presents an additional limitation to designed data sources; for example, the first releases from latest National Census 2021, which was conducted in August 2021, are planned for June 2022 [83]. Other designed data sources, which hold data from a smaller population and on fewer indicators release results more frequently [54].

The age of data collected in organic data sources is dependent upon a consumer’s contact with the organic data system. ieMR data is collected every time a person presents to the digital public hospital system in Queensland (70% of services) however the frequency of this contact cannot be predicted. Similarly, mHealth data is collected as often as the individual engages with the digital health wearable by automatic data collection or manually entering data into a mHealth application. Information about the age of data in Google Maps is inconsistent; some sources claim it is updated ‘every minute of every day’ [84] while others that it occurs every 1–3 years [85]. The Crime Map is updated in near real-time (daily). Billing/claims data is relatively recent (can be viewed for the previous month) however harvesting it requires thorough knowledge of item numbers- their description and how they can be used by health professionals.

#### Granularity

Across the designed data sources, National Census, which collects data from the people in Australia on the Census night, holds rich information on each social sub-determinant of obesity [53]. Data is available for Census-defined geographies from Australia down to Statistical Area Level 1 (‘mesh blocks’ that contain ~ 200–800 people) [86]. For example, National Census data on housing is presented as ‘dwelling structure’ (dwelling count as occupied, dwelling type, number of bedrooms, tenure) and ‘household composition’ (family, single, or group households) [87]. Designed data sources covering the state of Queensland represent a relatively narrow population coverage and scope (e.g. 12 500 adults and parents of 2 500 children (5-17 years) (stratified for male/female) on selected risk factors, e.g. smoking, BMI, health and wellbeing, physical activity, alcohol consumption or nutrition) [58]. Longitudinal studies [56, 60] report on the status and temporal change in social, economic and child development data of their nationally representative sample of 17 000 adults and 10 000 children, respectively.

There is enormous potential for organic data to be highly granular and this largely depends on the information entered to each data source. ieMR data is available for each individual who presented to a public hospital however data granularity would only be at a non-identifiable (suburb or greater) level. Whilst useful for point-of-care treatment and management, some clinical data from the ieMR may not be useful for improving population health (e.g. blood glucose levels, respiratory rate). Access to individual data from digital health wearables may be warranted depending on research study design and data owners’ approval. For example, Fitbit allows daily summary and intraday data (finer granularity of data) to be extracted for an individual account that has been a client ID [81]. Google Maps data can provide a view of a single postal address (residential or business), green space and street view, and offer a map or satellite view as well as street view images. The user can also change the level of magnification depending on needs. Online Crime Map allows to view offences (by type) for a specific location (suburb, postcode, Queensland Police Service Division, Neighbourhood Watch), data range (from yesterday to up to 5 years ago), offence type (detailed list) and time of day. Maps are displayed as cluster or heatmap.

Billing/claims data is the least granular organic data source [36, 37]. Data is available for item numbers or group of item numbers, services or benefits per State/Territory for month/s, periods, financial or calendar years. Data is only presented at a national level and cannot be granularized to individual states (e.g. Queensland).

#### Data coverage and gaps

Figure 1 presents each designed and organic data source (*n* = 21 with complete information available) and colour-codes their coverage of Level I determinants of obesity. Supplementary file 3 contains a more granular table representing each data source and their specific coverage of Level I and II determinants of obesity. The availability of data on determinants of obesity must be interpreted with caution. Sample sizes vary significantly across data sources and while some sources. For example, one designed source (longitudinal study [60]) covers all (10) social, 10 out of 14 biomedical and seven out of eight behavioural sub-determinants, the sample size is small (*n* = 10 000) while one organic source (ieMR) holds data on eight out of 10 social, 13 of 14 biomedical and five of eight behavioural Level II determinants but its sample size is approximately 5 M and is routinely collected.

##### Social determinants

Twenty-five designed and one organic data source contain data on one to all (10) social Level II determinants. Data on three Level II determinants is available in more than ten sources: ethnicity in 19 sources, employment in 13 and education in 12 data sources. Eight data sources exist for housing and domestic and personal safety data, seven for marital status, six for income, five for family structure/size, four for religion and two for child development. One longitudinal study contains data on all social Level II determinants in a small sample size (~ 10 000 persons) [60]. The National Census and ieMR hold data on eight social Level II determinants in a large sample size (~ 24 M for Census and 5 M for ieMR) but only ieMR data is routinely collected in real-time.

##### Biomedical determinants

Twenty-eight designed and three organic data sources contain data on one to 13 out of 14 biomedical Level II determinants of obesity. Most biomedical data is concentrated to age (*n* 25 sources) and sex (*n* 24 sources). Eleven data sources (nine designed and two organic) exist for disability, nine for weight, height, weight status (seven designed and two organic), eight each (six designed and 2 organic) for BMI and mental health, six for heart health and blood pressure (three designed and three organic), four each for birth weight (three designed and one organic) and chronic diseases (two designed and two organic), three for medication (one designed and two organic), two each for waist circumference (designed) and maternal weight status (BMI) (one designed and one organic) and one each for rapid infant weight gain (organic) and family medical history (organic). One organic source (ieMR) contains data on 13 of the 14 biomedical Level II determinants.

##### Behavioural determinants

Eleven designed and three organic data sources contain data on one to seven out of eight behavioural Level II determinants. Data on physical activity and smoking is available in nine sources (seven designed and two organic). Eight data sources exist for dietary patterns (designed) and alcohol consumption (seven designed and one organic). ieMR data on dietary patterns may be available in non-structured (free text) format (clinician notes). Breastfeeding data exists in five sources (four designed and one organic) and sleep data can be found in four sources (two designed and two organic). One longitudinal source holds data on seven Level II determinants [60]. One organic source (ieMR) contains data on five level II determinants.

##### Environmental determinants

Eighteen designed and three organic data sources hold data on one to five out of ten environmental Level II determinants. Eighteen sources (16 designed and two organic) contain data geographical location (rurality). Four sources exist on transport system (three designed and one organic) and two each for food availability (one designed and one organic), access to technology/internet connection/connectivity (two designed), green and open spaces (one designed and one organic) and geographical location/crime (one designed and one organic). Data on time commuting, access to health services and density and type of food outlet exist in one source each. There were no data sources identified for residential density. One designed source (longitudinal study) [60] and one organic source (Google Maps) contain data on five Level II determinants each.

##### Commercial determinants

Six designed and one organic data source contain data on one or two out of six commercial Level II determinants. Seven sources exist on affordability (six designed and one organic) and two on price index (designed).

## Discussion

### Main findings

This scoping review identified 33 primary sources of designed data and five primary sources of organic data relevant to the five determinants of obesity (social, environmental, behavioural, biomedical and commercial) that can be used as the foundation to advancing PPH for obesity using Queensland, Australia as a globally-relevant use case.

We found four key findings relevant to our aims:

1. Designed data sources dominate the prevention and public health sector relevant to obesity but they are fragmented. There is no single source of truth for these sources. They are highly accessible but are often outdated, capture small sample sizes (< 100 000) relative to big, organic data sets (> 1 million participants), become redundant quickly (as they are sometimes released years after initial collection) and vary in granularity.
2. Data on social, biomedical and behavioural determinants of obesity exists across multiple designed data sources, primarily surveys, and in one organic source (ieMR [74, 80]); however, most biomedical data is age or sex only. Data on the environmental and commercial determinants of obesity was sparse and interpreted as low quality.
3. Designed data sources lack comprehensive, rich, granular and timely data that is required for preventive and public health decision-making for obesity. These sources often report data at a large geographic level (e.g. National or State) and use different questions/indicators to report on the same sub-determinants of obesity making it difficult to compare and aggregate data.
4. Public health agencies and leaders must prioritise ‘unlocking’ and operationalising organic data—complemented with designed data—to achieve high-value data-driven PPH for obesity and other NCDs.

### Analysis of current state

Currently, designed data sources are the cornerstone of public health decision making. Our results demonstrated a strength and significant weakness in the ubiquity of designed data – data on all determinants of obesity is mostly publicly available but is concentrated towards low contemporaneity and small sample size (Fig. 1). We found designed data to be fragmented across more than 30 sources that are infrequently updated (> 2y), often representing a small (< 100 k) population and often taking months to years to become available after collection. Designed data sources may be well-suited to capturing social (e.g. housing, education) and environmental (e.g. built infrastructure, walkability) data as their indicators are relatively static over time. Biomedical (e.g. infant weight gain, pre-pregnancy BMI), behavioural (e.g. physical activity, dietary patterns) and commercial (e.g. purchasing behaviours, advertisements) data has higher utility when routinely collected and analysed in near or real-time but requires new data-driven public health digital infrastructure.

It is difficult to count preventive impact and thus make prevention ‘matter’ without an agreed set of authoritative and contemporaneous data [4]. Data mapping for improving NCD-related population health has been performed in Maryland, USA; Hatef et al. [88] identified and evaluated national and local data sources to develop a population health measurement framework. Sample indicators included BMI, fruit and vegetable consumption, median household income, levels of housing affordability and current smoking status. The authors found diverse sources of designed (e.g. Youth Risk Behaviour Surveillance System, Census) and organic (e.g. electronic health record, health information exchange) data; however, recognised innate challenges to using designed data for advancing data-driven public health, including their small sample sizes and potential for linkage across sectors [88].

### Towards the future state – precision public health

Data becomes an asset when it can be transformed into information that is usable for decision-making. Organic data can unlock new and contemporaneous information that has not traditionally been accessible to public health practitioners, policymakers, recipients (consumers, communities, public) as data is routinely collected, processed and available in near real-time, as opposed to months or years after collection. Organic data arising from the digital transformation of the acute healthcare sector (primarily via the EMR) has already transformed acute care [10, 11]. Rich and contemporaneous data is now available for every patient, every time and in real-time. This capability is being used to establish a learning health system where all data entered is reused to improve the care of subsequent patients [89].

The potential benefits of using organic data for public health must be weighed against the technical challenges associated with capturing, integrating and processing such data, specifically volume, veracity, variety and velocity, and social challenges, such as ethical use and ownership of consumer health data [90]. The volume of organic data grows exponentially and its structured and unstructured data fields are subject to biases of the populations that interact with them, such as critically ill patients in hospital (EMR) or active, healthy persons tracking their activity (wearables). There are unresolved ethical challenges of using organic or ‘big’ data, such as governing an informed consent process, ensuring actions seek to reduce rather than widen health inequities, and redefining what ‘privacy’ means in the context of dynamic, real-time and granular health data [90, 91]. Designed data, for example, is ethically and socially accepted for routine decision-making at an organisational, government or research level; it has been declared as an asset for that specific purpose. Adequate steps to address these challenges must underpin a strong overarching message – that organic data is an untapped asset and there is significant opportunity cost of *not* using this data to transform and improve public health.

Complementary use of organic and designed data can be a powerful transitional step for improving the precision of public health decisions and policy in the short- and medium-term. In the context of our results, there would be strength in mobilising EMR data in Queensland and aggregating this organic data with high quality designed sources, such as the national Census [53] and Early Childhood Education and Care Census (ECEC) [72]. Clinical EMR data could be used to geotag hotspots of unhealthy weight trajectories in childhood and ECEC data overlayed to contextualise social position and education. This intervention example aligns with a PPH future and could help to inform new organisational priorities, policy change and resource allocation for preventing childhood obesity. A PPH future is a new way of working and will generate a disruptive shift in public health organisational and practitioner culture [92]. Digital infrastructure must be capable of capturing, integrating and processing near real-time organic and designed data for NCDs across multiple (likely heterogenous) sources [90]. A new era of public health practitioner may require additional digital, data and analytics training to correctly analyse, interpret and action rich, contemporaneous information with greater agility [92]. The inherent role of a public health practitioner may advance from long-term planning (based on retrospective data), to reactive planning (based on contemporary data). A suite of evidence-based preventive interventions may be available to an agile, reactive public health system to ‘push’ to at-risk populations as needed and evaluate impact. New public health workflows will require a responsivity comparable to a health service as risk-based hotspots require timely preventive ‘treatments’ to address risk. For example, pre-pregnancy weight status may be geotagged and aggregated with organic behavioural data (e.g. smoking, physical activity) to direct pregnancy-focused lifestyle education programs and social media messaging in those hotspot areas. Digitally-driven feedback loops can subsequently enable a virtuous learning cycle of prevention – a learning *public* health system [9].

The European Public Health Association’s recent (2019) vision of public health digitisation proposes digital transformation of public health will enable personalisation, precision, automation, prediction, data analytics and interaction [92]. Robust cross-sectoral partnerships will generate faster and more sustainable change towards this vision as data is required from all sectors (especially those outside of health) and PPH requires skills beyond a traditional public health practitioner. A strength of traditional public health is the rich community engagement – this must remain a core pillar of PPH. The conversation must shift towards ethical use of organic data for population health decision-making, how to empower communities with data, and leveraging their lived experienced to unlock the human context behind a community’s data (i.e. the qualitative story behind the quantitative numbers) to ensure appropriate interpretation and action.

### Strengths and limitations

There are several strengths to this review. PPH must start with evidence mapping to understand the current state of data availability and usability. Our review provides the first synthesis of designed and organic data usable for PPH of NCDs, offering Queensland, Australia and obesity as our use cases. We employed an iterative, comprehensive search strategy to systematically map designed and organic primary data sources usable for PPH of obesity. In lieu of an evidence-based tool to quantitatively appraise the quality of designed and organic data sources, we performed a detailed qualitative quality appraisal of each primary data source.

This review has several limitations. Due to our scoping design we may have missed sources of designed and organic data. One example of potential missing data is from the exclusion of relevant research trials. Large-scale trials may provide new insight into data gaps that exist with current sources of designed and organic data and could be included in future reviews with a highly specific literature search strategy. Our review only provided details about Level I and Level II determinants of obesity rather than specific indicators. We were unable to provide a quantitative quality assessment of primary data sources and so it is difficult to objectively compare data source quality.

## Conclusions

This scoping review presents a comprehensive map of primary data sources available to advance PPH via data-driven decision-making for public health professionals, policymakers, health system managers and planners, and prevention organisations in Queensland, Australia. We provide a globally re-usable framework for identifying, collating and appraising primary data sources that can be translated to communicable and NCDs by international public health agencies and health systems. Organic data will provide the foundation for PPH – organisations must address the short-term technical challenge of using organic data harmoniously with designed data, building digital infrastructure for high-quality organic data and addressing the ethical and social implications of using routinely collected consumer-centred data to realise this globally shared public health vision.

## Supplementary Information

**Additional file 1.**

**Additional file 2.**

**Additional file 3.**

## Data Availability

All data generated or analysed during this study are included in this published article and its supplementary information files.

## Abbreviations

BMI: Body mass index
EMR: Electronic medical records
ieMR: Integrated-electronic medical record
IoT: Internet of Things
MHR: My Health Record
PCC: Population, Concept and Context
PHR: Personal health records
PPH: Precision public health
PRISMA-ScR: Preferred Reporting Items for Systematic Reviews and Meta-Analyses extension for Scoping Reviews
RCT: Randomised controlled trial
SDAC: The Survey of Disability, Ageing and Carers
SME: Subject matter expert
