# Supplementary material for: Data sources for precision public health of obesity: a scoping review, evidence map and use case in Queensland, Australia
Source: BMC Public Health. 2022 Mar 24;22:584. doi: 10.1186/s12889-022-12939-x (PMC8953390; doi:10.1186/s12889-022-12939-x)
Supplement: Supplementary file 2 — Additional file 2. [file 12889_2022_12939_MOESM2_ESM.docx]

# Supplementary file 2: Sample search strategies

**Sample search – Google Scholar**

Search terms: ‘obesity and ‘Queensland’

Filter: 2000-2021

Results: 27100

**Sample search – Google**

Search term: Obesity Queensland

Language: English; Region: Australia; site or domain: .gov.au; File type: Adobe Acrobat PDF (.pdf)

Results: 16200

**Database searches using search terms:** ‘obesity prevention’ OR (obesity AND prevention) AND (Queensland OR QLD)

Embase Search:

Search/all fields: obesity AND prevent* AND (Queensland OR QLD); Results: 1525

Limit 1: Publication years 2000-2021; Results: 1514

Limit 2: Title, abstract, author keywords; Results: **34**

Scopus Search:

Search/All fields: obesity AND prevent* AND (Queensland OR QLD); Results: 9115

Limit 1: Publication years 2000-2021; Results: 9019

Limit 2: Title, abstract, author keywords; Results: **46**

WOS search:

Search/All fields: obesity AND prevent* AND (Queensland OR QLD); Results: 1306

Limit 1: Publication years 2000-2021; Results: 1294

Limit 2: Topic (Title, abstract, author keywords); Results: **39**

**Scopus database search using various search terms:**

Search: hous* AND Queensland 2000-present; title, abstract, keywords; Results: 879

Search: employment AND Queensland 2000-present; title, abstract, keywords; Results: 503

Search: income AND Queensland 2000-present; title, abstract, keywords; Results: 344

Search: education AND Queensland 2000-present; title, abstract, keywords; Results: 2742; added AND social; Results: 515

Search: ‘food availability’ AND Queensland 2000-present; title, abstract, keywords; Results: 171

Search: ‘food security’ AND Queensland 2000-present; title, abstract, keywords; Results: 36

Search: ‘marital status’ AND Queensland 2000-present; title, abstract, keywords; Results: 60

Search: (family AND (size OR structure)) AND Queensland 2000-present; title, abstract, keywords; Results: 343

Search: ethnicity AND Queensland 2000-present; title, abstract, keywords; Results: 125

Search: sleep AND Queensland 2000-present; title, abstract, keywords; Results: 125

Search: ‘birth weight’ AND Queensland 2000-present; title, abstract, keywords; Results: 175

Search: (‘physical activity’ OR exercise) AND Queensland 2000-present; title, abstract, keywords; Results: 518

Search: smoking AND Queensland 2000-present; title, abstract, keywords; Results: 349

Search: (alcohol AND (intake OR consumption OR use)) AND Queensland 2000-present; title, abstract, keywords; Results: 205
